# Dermatologic complication following axillary cannulation for aortic dissection repair

**DOI:** 10.1002/ccr3.1969

**Published:** 2019-01-09

**Authors:** Raisa D. Nguyen, Bryan J. Hierlmeier, Lakshmi N. Kurnutala

**Affiliations:** ^1^ Anesthesia University of Mississippi Medical Center Jackson Mississippi

**Keywords:** axillary artery cannulation, cardiopulmonary bypass, compartment syndrome, complications, hyperperfusion

## Abstract

It is important to be aware of complications associated with axillary artery cannulation, especially the more common ones that can compromise limb integrity. Pulse oximeter and arterial line placed on the right upper extremity can aid in perfusion of the right arm during right axillary artery cannulation.

## 1 INTRODUCTION

The axillary artery has become a popular site for arterial cannulation during cardiopulmonary bypass (CPB) when the ascending aorta cannot be used due to atherosclerosis, aneurysm, or dissection.^1^, ^2^, ^3^, ^4^, ^5^ We present a patient who developed widespread edema, bullae, and hyperperfusion syndrome of her right upper extremity (RUE) following right subclavian artery cannulation for emergent repair of a type A aortic dissection.

## 2 CASE DESCRIPTION

An 80‐year‐old Caucasian female with history of hypertension and chronic back pain presented for emergent repair of a 7.2 cm aneurysm of the ascending aorta with Stanford classification type A dissection. Because the ascending aorta was unsuitable for arterial cannulation, the surgeon elected to perform axillary cannulation via the right subclavian artery with side graft anastomosis. The patient arrived to the operating room (OR) with nicardipine and esmolol infusions running through an 18‐gauge peripheral intravenous (IV) line in the right antecubital (AC) fossa. Prior to induction of anesthesia, we disconnected the infusions from the right AC and administered medications though an 18‐gauge IV in the left forearm. The patient also had a left radial arterial line (AL), and we placed the pulse oximeter and noninvasive blood pressure (NIBP) cuff on the RUE. After intubation, we placed a right radial AL, and the surgeons placed a left femoral AL. All arterial pressures correlated closely. Additionally, a 9‐French central line was placed in the right internal jugular vein. The patient was cleansed and draped for surgery with her arms tucked to her sides.

Shortly after the procedure began, the right radial AL tracing went flat, and the pulse oximeter waveform was lost. We attributed this to the surgeon partially clamping the right subclavian artery in preparation for arterial cannulation. We switched the pulse oximeter to the left hand and relied on the left radial and femoral AL for pressure readings. Just prior to arterial cannulation, we noted that the right radial pressure returned, although about 20 points lower than the left radial/femoral. Immediately after initiating CPB, the right radial mean arterial pressure (MAP) increased to 200 mm Hg, and left radial/femoral MAP decreased from 60 to 30 mmHg. The perfusionist alerted the surgeon about the high line pressures and decreased CPB flows. After a brief attempt to troubleshoot and adjust the cannula with little improvement in pressure or flow, the surgeon proceeded with the operation. Over the next few minutes, the left radial/femoral MAP increased to 60 mmHg.

As deep hypothermic circulatory arrest (DHCA) was initiated, the right radial MAP decreased to 30 mmHg and left radial/femoral MAP decreased to 10. When CPB was reinstated, right radial MAP again increased to 200 mmHg. After 29 minutes of DHCA and 265 minutes of CPB, the patient was successfully weaned from CPB, and right radial MAP decreased to about 10 points lower than left radial MAP.

**Figure 1:**
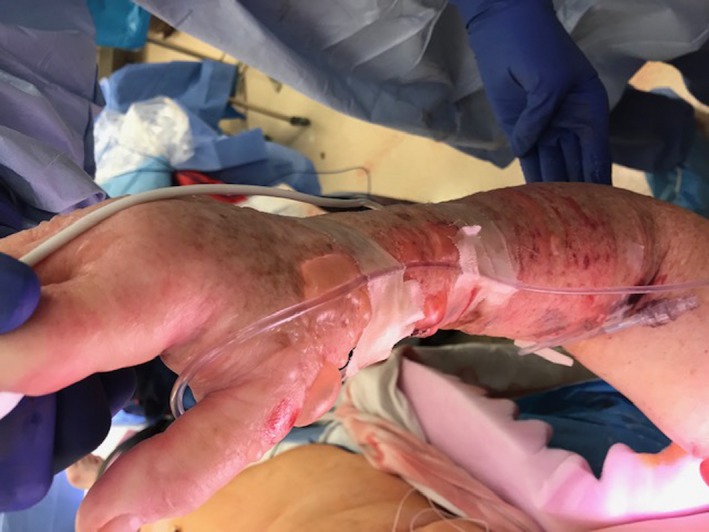
Right upper extremity showing blisters and bullae

**Figure 2:**
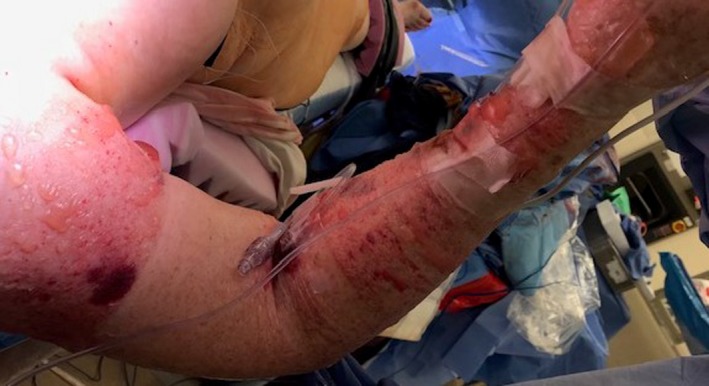
Right upper extremity showing blisters and bullae from the shoulder to the hand. The skin of the upper arm where the noninvasive blood pressure cuff had been placed was normal in appearance

Despite these issues, the surgery was otherwise uneventful. However, upon the surgical drapes being taken down, we noticed that the patient's RUE was swollen with blisters and bullae from the shoulder to the hand; yet, the skin of the upper arm where the NIBP cuff had been placed was normal as seen in figures. The IV in the right AC appeared to be infiltrated and weeping fluid even though we had not used it during the case and did not have any IV fluids attached to it (Figures 1 and 2). The IV and right radial AL were removed in the OR, and a Xeroform gauze dressing was applied to the RUE with the surgeons present. The intensive care unit nurses were instructed to elevate the arm and perform hourly neurovascular checks.

On postoperative day 1, the patient complained of tenderness and burning in the RUE, but she maintained adequate capillary refill, motor function, and sensation. Plastic Surgery was consulted to rule out compartment syndrome. They were unsure of the diagnosis but recommended nonoperative management and continued neurovascular checks. Eventually Dermatology was also involved, and they performed a punch biopsy of the patient's right dorsal hand. Their initial diagnosis was allergic contact dermatitis (ACD) due to the fact that the area of skin covered by the NIBP cuff was spared. However, the biopsy showed pauci‐inflammatory dermal‐epidermal blistering, which did not favor ACD. Direct immunofluorescence was also negative, ruling out localized pemphigus. Given the histologic findings, the final diagnosis was hydrostatic edema/bullae correlating with rapid edema during surgery. The patient continued to be managed nonoperatively with 1% triamcinolone ointment and gauze dressings, and within one month the blisters had completely resolved.

## 3 DISCUSSION

The axillary and femoral arteries are common sites for arterial cannulation during CPB when the ascending aorta cannot be used due to severe atherosclerosis, aneurysm, or dissection.^1^, ^2^, ^3^, ^4^, ^5^ However, which site offers the best operative and postoperative outcomes remains up for debate.^6^, ^7^ Axillary cannulation is often favored because of the lack of atherosclerosis of the axillary artery, reduction of atherosclerotic embolization, stroke, and retrograde dissection that may occur with femoral cannulation, and antegrade aortic and cerebral perfusion.^1^, ^2^, ^3^, ^4^ Two methods of axillary cannulation include direct cannulation of the artery and indirect cannulation via a side graft anastomosed to the artery “end‐to‐side.” Likewise, the subclavian artery may be cannulated in a similar fashion.^1^, ^2^ In this case, the surgeon performed a side graft anastomosis of the right subclavian artery.

Observed and theoretical complications of axillary cannulation include arterial/aortic injury or dissection, small vessel size leading to cannulation problems or inadequate CPB flow, malperfusion, arterial thrombosis, and ipsilateral limb complications (hyperperfusion syndrome, compartment syndrome, ischemia, bleeding, wound infection, and brachial plexus injury).^1^, ^2^, ^8^, ^9^ Hyperperfusion syndrome, defined as an edematous limb that is hyperemic and warm to the touch, is one of the most common complications associated with axillary cannulation,^8^ whereas thrombosis, wound infection, and brachial plexus injury are plausible but have not been reported.^2^ Most complications are seen with direct cannulation of the artery; however, malperfusion and hyperperfusion syndrome are more frequently seen with indirect cannulation.^1^, ^2^ In addition, indirect cannulation requires more time initially due to having to anastomose a graft to the artery (although it also simplifies decannulation), has more bleeding from an increased number of suture lines, may have leaking through the graft during CPB, and has a greater association with hyperperfusion syndrome in VA ECMO when compared to femoral cannulation.^1^, ^2^, ^8^

While our patient experienced hyperperfusion syndrome of her RUE, she did not develop the more serious sequelae of compartment syndrome or ischemia and was able to be treated with conservative management with no long‐term effects. The mechanism of hyperperfusion appears to be multifactorial: limb hyperemia due to arterial cannula flow, arterial inflow obstruction, and venous outflow obstruction.^8^, ^9^ Arterial obstruction can be caused by difficulties with construction of the side graft anastomosis, leading to a narrowed axillary artery lumen and preferential flow down the limb.^8^ Venous obstruction can be caused by bleeding around the cannula into the surrounding tissues thus creating a hematoma, direct compression of the axillary vein by an arterial or venous cannula, deep venous thrombosis, or external compression. We believe that our patient experienced a combination of arterial cannula obstruction, as evidenced by our extremely high arterial line pressures and decreased CPB flows, as well as venous outflow obstruction. Though we initially thought that the presence of the NIBP cuff on the upper arm acted as a protective tourniquet to the underlying skin by preventing fluid extravasation, it is more likely that the cuff contributed to external occlusion of venous return.^9^

## 4 CONCLUSION

Although there are many advantages to axillary artery cannulation over femoral cannulation, it also comes with its own problems. It is important to be aware of these complications, especially the more common ones that can compromise limb integrity. In our case, we were fortunate to have the pulse oximeter and right radial AL to aid in monitoring RUE perfusion; yet, we were not aware of the danger of having the NIBP cuff on the ipsilateral arm. In the future, we will avoid placing the NIBP cuff on the same side as the cannulation site, and we will be cognizant of the signs and symptoms of hyperperfusion or compartment syndrome.

## ETHICS APPROVAL

The patient gave permission for the authors to use the images and to publish the case report.

## CONFLICT OF INTEREST

None declared.

## AUTHOR CONTRIBUTION

RDN: participated in the case, wrote the original manuscript, edited and reviewed the final manuscript. BJH: participated in the case, edited and reviewed the final manuscript. LNK: edited and reviewed the final manuscript.
